# Aortic Dissection With Complete Occlusion of Left Main Coronary Artery Presenting as Acute ST-Segment Elevation Myocardial Infarction

**DOI:** 10.7759/cureus.15595

**Published:** 2021-06-11

**Authors:** Ajay Pratap Singh, Vatsal Kayal, Ranjit K Nath

**Affiliations:** 1 Cardiology, Atal Bihari Vajpayee Institute of Medical Sciences and Dr. Ram Manohar Lohia Hospital, New Delhi, IND

**Keywords:** aortic dissection, coronary angiography, stemi, myocardial infarction, left main stem occlusion

## Abstract

Acute Aortic Dissection (AAD) is a life-threatening condition, which presents with a wide variety of symptoms ranging from being asymptomatic to sudden cardiac death. A retrograde extension of AAD can lead to partial or complete occlusion of coronary vessels, leading to an exceedingly rare presentation in the form of acute Myocardial Infarction (MI). A prognosis of AAD depends on prompt diagnosis and urgent surgical intervention to re-establish coronary blood flow. Here, we report a case of AAD, presenting as acute anterolateral wall MI, due to total occlusion of the left main coronary artery.

## Introduction

Acute Aortic Dissection (AAD) is characterized by the rapid development of an intimal flap separating the aortic lumen into true and false lumina. The dissection can spread in an antegrade or retrograde fashion, involving side branches, and cause complications such as malperfusion syndrome due to the dynamic or static obstruction (from coronary to iliac arteries), tamponade, or aortic insufficiency [1]. Aortic dissections are classified according to their anatomical location using the Stanford classification. The fundamental distinction is whether the dissection is proximal (Stanford A: involving the aortic root or ascending aorta) or distal (Stanford B: beyond the left subclavian artery) [2].

Around 0.5% of patients presenting to an emergency department with chest or back pain suffer from aortic dissection [3]. Acute Myocardial Infarction (MI) due to hypoperfusion of the coronary artery is an exceedingly rare finding in AAD, but it is potentially fatal. The incidence of MI among AAD patients is 1-2% due to the compromise of the coronary ostium by the hematoma or intimal flap [3]. The main challenge in AAD management is having the appropriate clinical suspicion and action to pursue a diagnosis and the corresponding therapy [4]. In up to 40% of cases, AAD is misdiagnosed in the emergency room (ER) due to its clinical and epidemiological overlap with acute MI [5]. Here, we report a case with a Stanford type A aortic dissection, presenting as an acute anterolateral wall MI due to left main coronary artery (LMCA) obstruction.

## Case presentation

**Figure 1:**
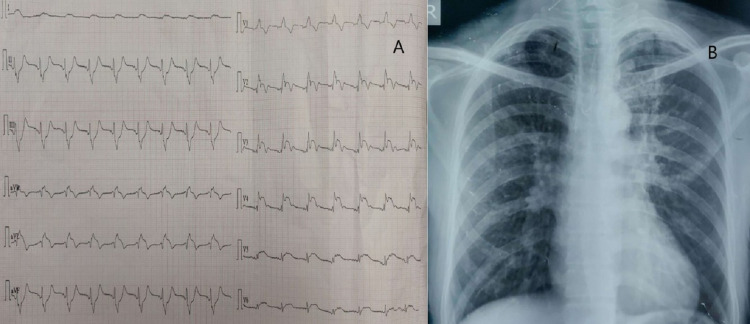
[A] 12-lead ECG showing ST-segment elevation in lead I, aVL, and V2 to V6 along with reciprocal ST-depression in lead II, III & Avf, [B] Chest X-ray PA view. ECG- electrocardiogram, PA- posteroanterior

A 45-year-old Asian male, with a 20-year history of smoking and poorly controlled hypertension, presented to the ER with a history of severe central chest pain, radiating to the left shoulder and back for the previous 4 hours. The patient was severely diaphoretic and extremely distressed due to the pain. On presentation, his vitals showed that he was hypotensive (blood pressure of 80/46 and 84/40 mm Hg in the right and left arm, respectively) and tachypneic. His pulse was regular and hypovolemic, with no radioradial or radiofemoral delay. There was no cardiac murmur and the bilateral lung fields were clear. The remainder of the physical examination was normal. A 12-lead electrocardiogram (ECG) showed extensive ST-segment elevation in lead I, aVL, and the chest leads (V2 to V6) with reciprocal ST-depression in inferior leads (II, III, aVF) (Figure 1A). Blood parameters were within normal limits and troponin T was positive (qualitative). A chest X-ray was normal (Figure 1B) and two-dimensional echocardiography (2D-echo) screening showed hypokinesia of anterior and anterolateral segment with apical hypokinesia. The left ventricular ejection fraction (LVEF) was estimated to be 30-35%, with normal valvular functions. There was no evidence of a dissection flap or dilatation of the aortic root and ascending aorta. Considering all these findings, a diagnosis of acute anterolateral wall MI was made, and the patient was prepared for primary percutaneous coronary intervention (PCI).

**Figure 2:**
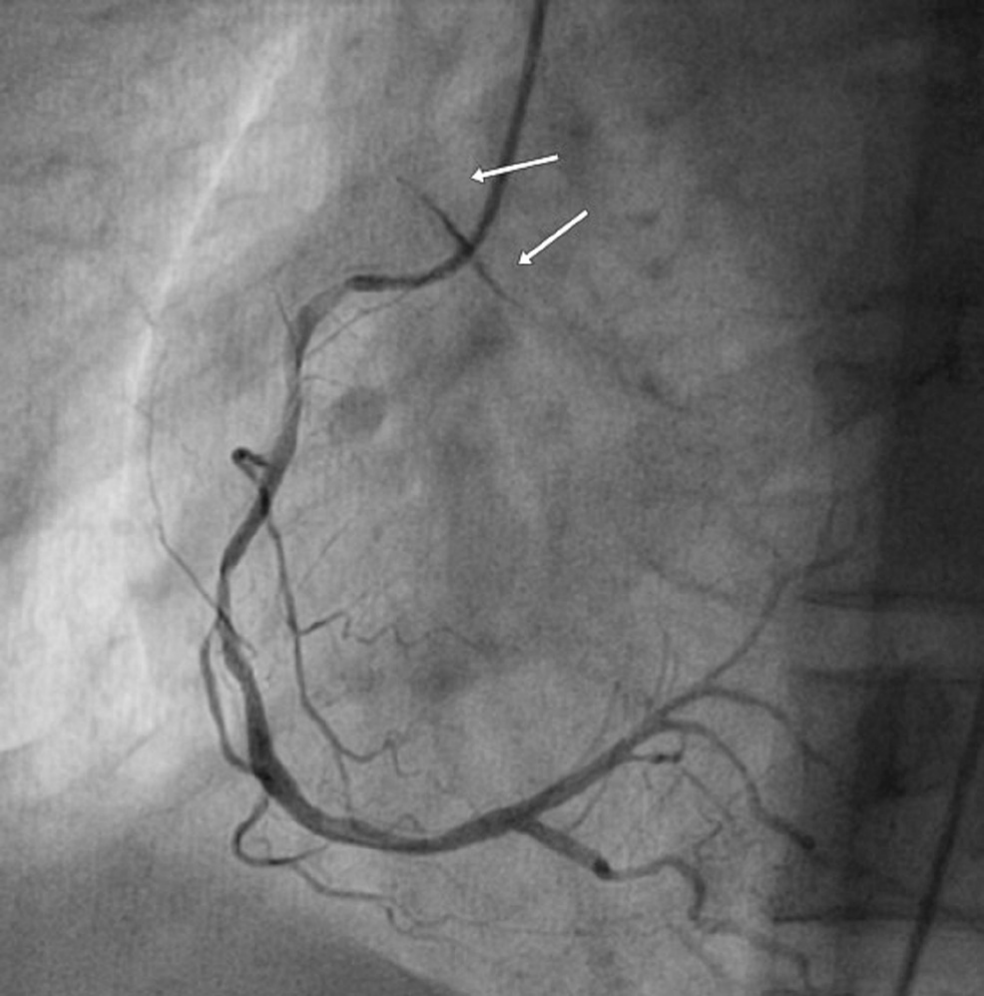
Coronary angiogram of right coronary artery showing proximal plaque and mid-segment mild disease, contrast staining the root of the aorta can be seen (white arrows) suggestive of aorto-ostial dissection.

He was administered aspirin 300mg, ticagrelor 180mg, and immediately shifted to the cardiac catheterization laboratory. An intravenous injection of unfractionated heparin 3000 IU was given in the lab and femoral access was preferred, considering the hemodynamic parameters of the patient. The right femoral artery was cannulated with a 7- Fr sheath and the right coronary artery was cannulated using a 6-Fr diagnostic Judkins Right (JR 4.0) catheter (Medtronic, Minneapolis, MN). The angiogram revealed a 70% lesion in the middle segment of the right coronary artery (RCA), but there was staining of the aortic sinus involving the RCA ostium on both sides (Figure 2).

**Figure 3:**
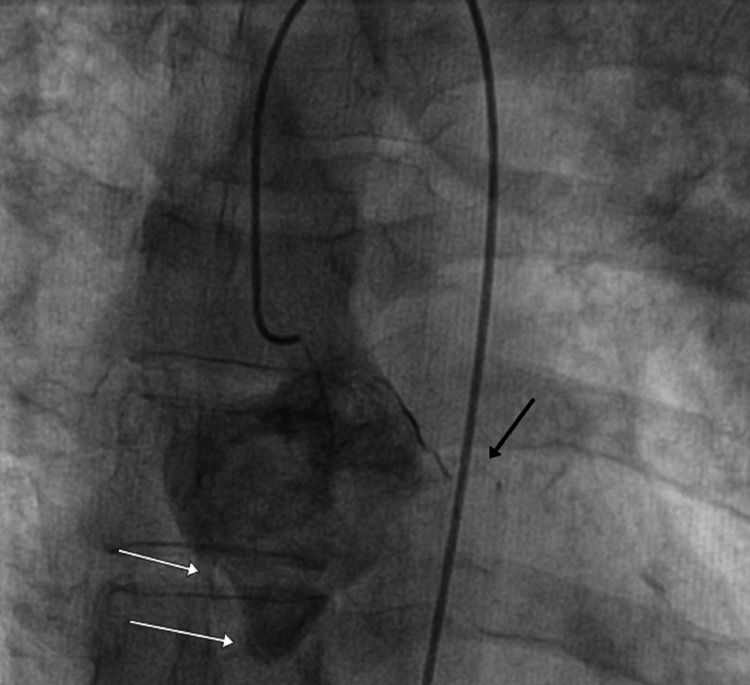
Non-selective coronary angiogram of a left-sided system. Dilated aortic root along with patchy staining of the left main coronary artery (black arrow) and contrast staining of the root of the aorta are visible. A dissection flap can be seen at the root of the right coronary artery (white arrows).

This immediately pointed toward the presence of an aorto-ostial dissection involving the coronary ostia. We then took an extra backup 3.5 EBU guiding catheter (Cordis, Hialeah, FL) for cannulation of the left main coronary artery (LMCA), but despite multiple attempts, the left system could not be cannulated successfully. At this point, we decided to take a non-selective shot using the diagnostic JR, high in the aorta, which showed faint antegrade staining of the LMCA without visualization of the left anterior descending (LAD) or the circumflex artery (LCx) (Figure 3).

The patients’ hemodynamic parameters deteriorated, so vasopressor support was initiated. An urgent call for the attending cardiothoracic and vascular surgeon was made to consider the surgical repair of the dissection and re-establishment of coronary blood flow.

**Figure 4:**
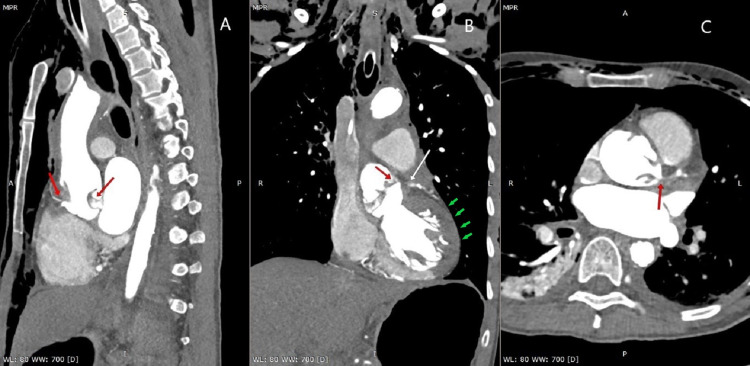
Contrast CT aortogram [A] sagittal view showing the dissection flap originating anteriorly near the right coronary artery ostium (red arrow) and extending posteriorly and attached near the LMCA ostium (red arrow), [B] coronal section showing the dissection flap (red arrow) causing complete occlusion of the LMCA (white arrow) with nonenhancement of the myocardium (green arrows), [C] cross-sectional view showing the dissection flap obstructing the LMCA (red arrow) and faint filling of the branches of the LMCA. LMCA- left main coronary artery, CT- Computerized tomography

In the meantime, the patient was shifted for an urgent contrast-enhanced computerized tomography (CT) of the aorta, which confirmed our diagnosis of aortic dissection and revealed a curvilinear dissection flap in the ascending aorta, which extended inferiorly up to the aortic sinus. Anteriorly, the flap was attached to the aortic sinus distal to the right coronary ostia while it was attached to the site of LMCA ostium posteriorly (Figure 4A), occluding its lumen with minimal patchy opacification of the left coronary system via the LAD and LCx (Figure 4B, 4C).

**Figure 5:**
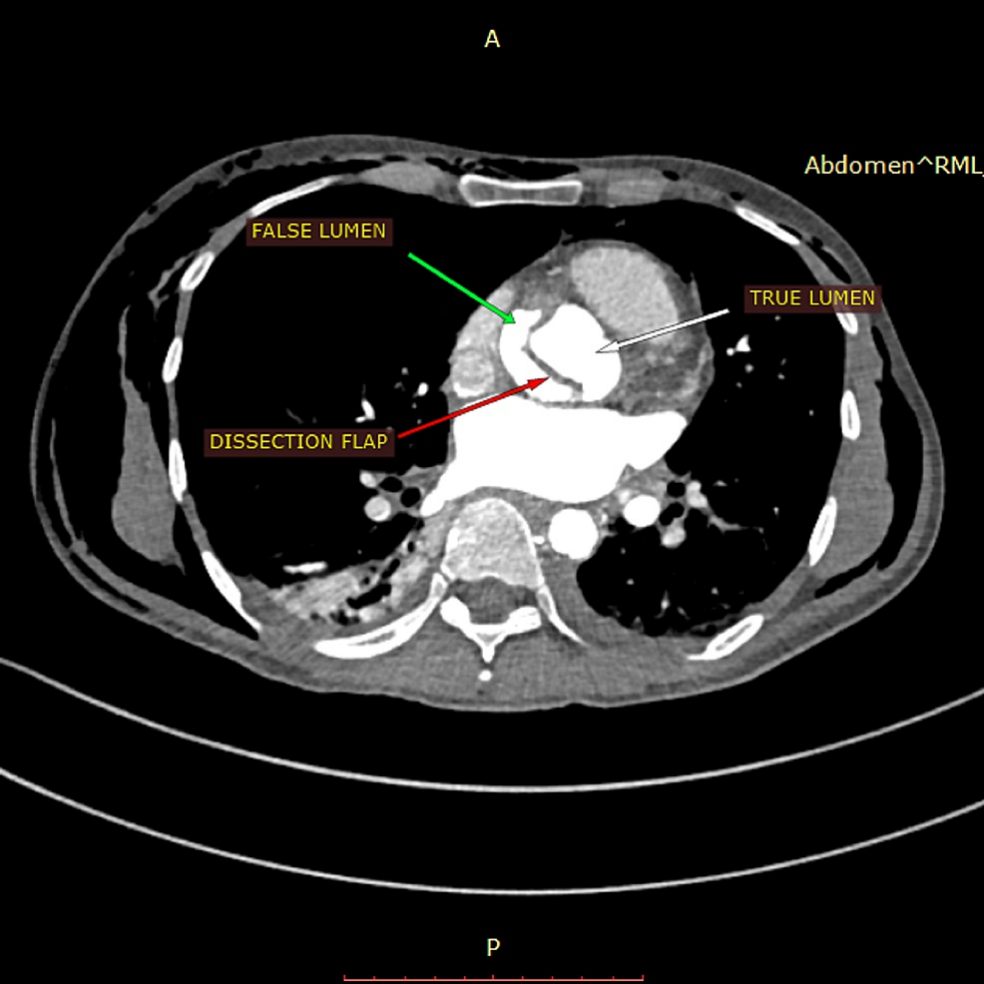
Dissection flap dividing the lumen of the aorta in the true and false lumen.

A dissecting flap divided the lumen of the aorta into true and false lumina, with evidence of thrombus within the false lumen (Figure 5).

There was nonenhancement of the anterolateral myocardial walls suggestive of ischemic changes (Figure 4B). The patient was shifted for urgent surgical correction but ultimately succumbed due to cardiopulmonary arrest during surgery.

## Discussion

Type A Stanford AAD with occlusion of the left main coronary trunk is a lethal complication, which requires urgent surgical intervention to guarantee the patient's survival. A history of systemic hypertension is found in up to 72% of patients, which is by far the most common risk factor. Other factors associated with AAD are atherosclerosis, prior cardiac surgery, a known aneurysm, known connective tissue disorder, bicuspid aortic valve, and prior aortic surgery [6].

The diagnosis of AAD begins with a clinical suspicion, which is the most crucial step in the work-up of this catastrophic condition. AAD presents with a wide spectrum of manifestations; typical features include the acute onset of chest and/or back pain of blunt, radiating, and migrating nature, but these are often absent. In one large study, the classic tearing or ripping type of pain was reported by only 51% [6] of patients. Shock in patients with aortic dissection is caused by acute severe aortic regurgitation, aortic rupture, cardiac tamponade, left ventricular systolic dysfunction, or bleeding from the rupture. The presence of pulse differentials is the most specific physical sign of aortic dissection, and it has been reported in 38% of patients with aortic dissection [6].

Despite major advances in the non-invasive diagnosis of aortic dissection and therapy, 28-55% of patients die without a correct antemortem diagnosis [6,7]. A battery of diagnostic tests can be used in the case of a clinical suspicion of AAD. Most importantly, the diagnostic study must confirm, or refute, the diagnosis of AAD, but unfortunately, no single test can predict it with certainty. Being the first and foremost investigation tool in an emergency, ECG has shown acute changes in up to 55% of patients [8] and includes ST-segment depression, T-wave changes, and ST-segment elevation. MI occurs in 1-2% of patients due to the compromise of the coronary ostium by the hematoma or intimal flap [3]. The classic radiographic sign that is suggestive of aortic dissection is the widening of the mediastinal shadow, which is seen in only about 56% of cases [8]. Transthoracic echocardiography has a sensitivity of 60% and a specificity of 83% for type A dissection [9], whereas transesophageal echocardiography (TEE) with color Doppler interrogation overcomes the limitations of transthoracic echocardiography, with a sensitivity of 94-100% for identifying an intimal flap and specificity ranging from 77-97% [10].

CT aortography provides complete anatomical information of the aorta, including branch vessel involvement, and enables visualization of the ostium and proximal part of both coronary arteries, with a sensitivity of 83-100% and a specificity of 90-100% for AAD. Conventional contrast aortography, an invasive procedure, is no longer required for diagnosing aortic dissection. The sensitivity and specificity of aortography are inferior to non-invasive imaging modalities. False negatives may occur if both the true and false lumen opacity appear equal with contrast, or if the false lumen is completely thrombosed [11]. MRI is a highly accurate diagnostic tool for the detection of AAD and has the highest accuracy, sensitivity, and specificity for all types of acute aortic syndromes, but it is used in <5% of patients in the International Registry of Acute Aortic Dissections (IRAD) due to issues with availability [12]. The use of more than one test provides a better predictive prediction for the detection of AAD. In a study by Thomas T et al. [12], an average of 1.8 diagnostic methods were utilized to diagnose aortic dissection.

Type A aortic dissection involving coronary ostia has a high mortality rate of 1-2% per hour after symptom onset [12]. Data from the largest registry of acute aortic dissection showed that, in the absence of immediate surgical repair, medical management is associated with a mortality of nearly 24% on day 1, 29% at 48 hours, 44% on day 7, and 50% after 2 weeks [13]. Even with surgical repair, in-hospital mortality rates are 10% at 24 hours, 12% at 48 hours, 16% at 7 days, and nearly 20% at 14 days [12].

Differentiating acute MI and AAD depends on a set of specific pointers ranging from the history and examination of the patient, as discussed earlier, the sensitivity and specificity of these pointers is low, hence we must rely on imaging to rule out similar conditions. As in this reported case, the patient history and a clinical examination failed to point toward the suspicion of AAD: the chest X-ray along with 2D-echo were also inconclusive, coronary angiography gave the first clue to suspect AAD, which was then confirmed by the contrast CT aortography. Similar cases have been reported in the literature where AAD was managed as an acute MI. In a review of 14 cases by Michael et al. [14], with Stanford type A dissection presenting as acute MI, 9 patients were managed with PCI, with a total of 4 deaths. In a report by Cyril et al. [15], AAD was diagnosed after successful PCI to LMCA using TEE. In another report by Ahmet et al. [16], despite the classical history of tearing chest pain radiating to the back, a CT aortography failed to recognize AAD involving the ascending aorta and the patient went for coronary angiography, which revealed normal coronaries. It was only on TEE where the dissection flap was identified as transiently obstructing LMCA ostium and was managed surgically. All these cases point toward the fact that no single imaging modality can diagnose AAD with utmost specificity; hence, we emphasize the use of more than two screening tests to improve the predictive power for diagnosing AAD. Regarding the management of AAD, urgent surgical intervention is the definitive treatment, but for patients with hemodynamic instability, as in our case, PCI has been reported as a bridging procedure before surgical correction and should be considered as a viable option, as reported by Amir et al. [17].

The take-home message from this case is that a carefully illustrated history and clinical examination may also miss the diagnosis of AAD; hence, the point of contact diagnostic non-invasive test should be implied with utmost sensitivity to screen this lethal disease, which often mimics acute MI, leading to significant misdiagnosis and potentially hazardous management with thrombolysis. The use of more than two screening imaging tests is immensely helpful in improving the predictive power to rule out AAD and there is a need for a streamlined facility and protocol-driven treatment strategies, such as those in MI or stroke, for the better diagnosis and prompt management of AAD.

## Conclusions

AAD mimics symptoms of many underlying pathologies. Given its low incidence, it therefore possesses a diagnostic challenge. Definite diagnosis relies on non-invasive imaging, which is also crucial to ensure prompt surgical intervention and avoid hazardous management approaches by wrongly thinking it is an acute MI.
